# Dysregulated Long Non-coding RNAs in Parkinson’s Disease Contribute to the Apoptosis of Human Neuroblastoma Cells

**DOI:** 10.3389/fnins.2019.01320

**Published:** 2019-12-13

**Authors:** Yun Fan, Jingyi Li, Qingmei Yang, Chengwu Gong, Hongling Gao, Zhijuan Mao, Xiao Yuan, Suiqiang Zhu, Zheng Xue

**Affiliations:** ^1^Department of Neurology, Tongji Hospital, Tongji Medical College, Huazhong University of Science and Technology, Wuhan, China; ^2^Department of General Surgery, The Second Affiliated Hospital of Nanchang University, Nanchang, China

**Keywords:** Parkinson’s disease, long non-coding RNAs, leukocytes, apoptosis, microarray

## Abstract

The molecular mechanism underlying Parkinson’s disease (PD), an increasingly common neurodegenerative disease, remains unclear. Long non-coding RNA (lncRNA) plays essential roles in gene expression and human diseases. We hypothesize that lncRNAs are involved in neuronal degeneration of PD. Using microarray, we identified 122 differentially expressed (DE) lncRNAs and 48 DE mRNAs between the circulating leukocytes from PD patients and healthy controls. There were 714 significant correlations (*r* ≥ 0.8 or ≤−0.8, *p* < 0.05) among the DE lncRNAs and mRNAs. Gene function and pathway analysis of the 48 DE mRNAs revealed biological pathways related to PD pathogenesis, including immune response, inflammatory response, MAPK, and Jak-STAT pathway. In a cohort of 72 PD patients and 22 healthy controls, the upregulation of four lncRNAs (AC131056.3-001, HOTAIRM1, lnc-MOK-6:1, and RF01976.1-201) in circulating leukocytes of PD patients were further confirmed. These lncRNAs were also upregulated in THP-1 cells, a human monocytic cell line, after inflammatory stimulation. Interestingly, the conditioned culture medium of THP-1 cells or 6-OHDA significantly increased the expression of these lncRNAs in SH-SY5Y cells, a human neuroblastoma cell line expressing dopaminergic markers. Importantly, overexpression of AC131056.3-001 or HOTAIRM1 increased baseline and 6-OHDA-induced apoptosis of SH-SY5Y cells. Taken together, we identified distinct expression profiles of lncRNA and mRNA in circulating leukocytes between PD patients and healthy controls. Dysregulated lncRNAs such as HOTAIRM1 and AC131056.3-001 may contribute to PD pathogenesis by promoting the apoptosis of dopaminergic neuron.

## Introduction

Parkinson’s disease is the most common neurodegenerative movement disorder affecting approximately 2% of people in the world (Wirdefeldt et al., 2011). Its clinical manifestations encompass motor symptoms such as rigidity, bradykinesia, and resting tremor (Goedert et al., 2013), and non-motor symptoms including sleep disturbance, hyposmia, constipation, and hallucinations (Schapira et al., 2017). The progressive degeneration and loss of dopamine neurons in pars compacta of the substantia nigra in the midbrain and the abnormal aggregations of α-synuclein are the key pathological changes of PD (Kalia and Lang, 2015). Apoptosis of dopaminergic neurons, neuroinflammation, mitochondrial dysfunction, oxidative stress, and alterations of the human microbiome have been implicated in the pathogenesis of PD (Olanow, 2007; Sampson et al., 2016). However, the essential molecular mechanism underlying PD remains largely unknown.

Long non-coding RNAs are a class of non-coding RNAs longer than 200 nucleotides without protein-coding potential (Kapranov et al., 2007). LncRNA had ever been thought as transcription noise (Birney et al., 2007). Recently, lncRNAs have been reported to play critical roles in gene expression, gene imprinting, chromosome conformation, cell differentiation, cell cycle, and apoptosis (Ponting et al., 2009; Rinn and Chang, 2012). Aberrant lncRNAs expression profiles were reported in human disease (Batista and Chang, 2013), including cancer (DasGupta et al., 2017), cardiovascular disorders (Bar et al., 2016), and diabetes mellitus (Knoll et al., 2015).

LncRNAs have been shown to be abundantly expressed in central neuron system (CNS) and play important roles in the development and disease of CNS (Mercer et al., 2008; Ng et al., 2013), including epilepsy (Lee et al., 2015), multiple sclerosis (Zhang et al., 2017), and ischemic stroke (Bhattarai et al., 2017). Moreover, lncRNAs were reported to be involved in the pathogenesis of neurodegenerative disease, including Alzheimer’s disease (AD), Huntington disease, and PD. BACE1 antisense RNAs (BACE1-AS) were overexpressed in the brain of AD, leading to the aggravation of AD pathology (Faghihi et al., 2008). LncRNA NEAT1 was shown to contribute to neuroprotection via increasing the neuron cell viability in Huntington disease (Sunwoo et al., 2017). The abnormal expression profiles of lncRNAs were found in the substantia nigra of PD patients (Kraus et al., 2017). However, the role of lncRNA in the pathogenesis of PD remains unclear.

Circulating immune cells are involved the pathogenesis of PD. It has been reported that both nitric oxide production and oxidative stress levels were significantly increased in circulating neutrophils of PD patients (Gatto et al., 2000; Vitte et al., 2004). Brochard et al. (2009) reported that infiltration of CD4^+^ lymphocytes into the brain contributes to neurodegeneration in a mouse model of PD. The recruitment of peripheral immune cells including monocytes, macrophages, and lymphocytes in the rat brain prior to neurodegeneration has also been observed (Harms et al., 2017). Therefore, we hypothesized that lncRNAs in circulating leukocytes from PD patients are altered and play a role in PD pathogenesis.

In this study, we profiled the expression of lncRNAs and mRNAs in circulating leukocytes from PD patients using microarray. We verified four upregulated lncRNAs in 72 PD patients and 22 healthy controls. In *in vitro* experiments, we examined the role of these upregulated lncRNAs in the apoptosis of SH-SY5Y cells.

## Materials and Methods

### Subject Recruitment and Blood Sample Collection

Blood samples were collected from 5 PD patients and 5 healthy controls for microarray, and from 72 PD patients and 22 healthy controls for quantitative PCR. All subjects were recruited at Tongji Hospital. The subject characteristics are summarized in Tables 1, 2. A neurologist specialized in movement disorders made the diagnosis of PD based on the International Parkinson and Movement Disorder Society (MDS) clinical diagnostic criteria for PD (Postuma et al., 2015). Exclusion criteria were as follows: (1) the presence of other neurologic illness or injury (traumatic brain injury, stroke, epilepsy); (2) the presence of chronic inflammatory disease; (3) the presence of previous malignancies or cardiac events; and (4) unstable psychiatric disorders such as schizophrenia or major depression.

**TABLE 1:** Clinical characteristics of PD patients and healthy controls for microarray.

		**WBC**	**RBC**	**Disease**		
	**Age**	**count**	**count**	**duration**	**H–Y**	**UPDRS-III**
	**(years)**	**(10^9^/L)**	**(10^12^/L)**	**(year)**	**grade**	**scores**
**PD**						
No. 1	63	6.33	4.27	5	2.5	46
No. 2	63	5.35	4.7	7	2.5	63
No. 3	66	4.49	4.25	2	2.5	19
No. 4	59	3.68	3.99	3	2.5	25
No. 5	58	8.57	4.05	3	2.5	27
**HC**						
No. 1	59	4.56	4.29	NA	NA	NA
No. 2	61	6.47	5.11	NA	NA	NA
No. 3	64	4.25	4.82	NA	NA	NA
No. 4	57	5.25	4.93	NA	NA	NA
No. 5	55	5.53	4.79	NA	NA	NA

*WBC, white blood cells; RBC, red blood cells; H–Y grade, Hoehn and Yahr motor grading; UPDRS-III scores, Unified Parkinson’s disease rating scale part III (UPDRS III) score.*

**TABLE 2:** Clinical characteristics of PD patients and healthy controls for quantitative PCR.

		**Healthy**	
	**PD patients**	**controls**	***p*-value**
Total number of subjects	72	22	NA
Male (%)	32 (44.4)	10 (45.4)	0.9335
Age (years)	58.86 ± 9.034	58.32 ± 7.409	0.7982
White blood cells count (10^9^/L)	5.289 ± 1.467	5.48 ± 1.074	0.5747
Red blood cells count (10^12^/L)	4.277 ± 0.4831	4.418 ± 0.3643	0.2102
Disease duration	4.41 ± 3.026	NA	NA
H-Y rank	2.419 ± 0.8223	NA	NA
UPDRS-III	34.02 ± 15.89	NA	NA

Blood samples were collected between 6 and 10 a.m. Red blood cells were removed immediately using red blood cell lysis buffer (Haoyang Biology, Tianjin, China). The leukocyte-enriched samples were immediately added into RNAiso Plus (TaKaRa, Dalian, China) and stored at −80°C.

### Microarray Analysis

Total RNA was extracted from circulating leukocytes using RNeasy Mini Kit (Qiagen, GmBH, Hilden, Germany) according to the manufacturer’s instructions. RNA was quantified using NanoDrop ND-2000 spectrophotometer (Nano-Drop Technologies, Wilmington, DE, United States) and RNA integrity was assessed using Agilent Bioanalyzer 2100 (Agilent Technologies, Santa Clara, CA, United States). Then, total RNA was amplified and labeled by the Low Input Quick Amp WT Labeling Kit (Agilent Technologies, Santa Clara, CA, United States). Cy3-labeled complementary RNA was hybridized with each slide using the Gene Expression Hybridization Kit (Agilent Technologies, Santa Clara, CA, United States). Then slides were scanned by Agilent Microarray Scanner (Agilent Technologies, Santa Clara, CA, United States). Data were extracted with Feature Extraction software 10.7 (Agilent Technologies, Santa Clara, CA, United States). Normalized signal values were converted into base-2 logarithmic values. We used a criterion of fold change ≥ 2 and *p* < 0.05 to identify DE lncRNAs and mRNAs between PD patients and healthy controls.

### Quantitative PCR

Total RNA was extracted from circulating leukocytes using RNAiso Plus (TaKaRa, Dalian, China) according to the manufacturer’s instructions. Total RNA was reverse transcribed into cDNA using PrimeScript RT Master Mix (TaKaRa, Dalian, China) according to the manufacturer’s instructions. Quantitative PCR was performed using SYBR Green Realtime PCR Master Mix (Roche, Mannheim, Germany) and the ABI ViiA7 QPCR System (Applied Biosystems, Carlsbad, CA, United States). Specific primers used for the reaction are as follows: AC131056.3 -001-F, 5′-aacagatagcccagggcatttt-3′, AC131056.3-001-R, 5′-ccca cgtcctcctcattcaca-3′; HOTAIRM1-F, 5′-gatttggagtgctggagcgaaga-3′, HOTAIRM1-R, 5′-gggttcaggcaaaacagacctc-3′; lnc-MOK-6:1-F, 5′-gtcaatttttctttcttctcttgc-3′, lnc-MOK-6:1-R, 5′-ctcctttattcttcgtt cctccaa-3′; RF01976.1-201-F, 5′-cttaatgctttcggacgggg-3′, RF01976. 1-201-R, 5′-gcgattcgtcctacgctcat-3′; DEFA4-F, 5′-tccaggcaagagg tcatgag-3′, DEFA4-R, 5′-cacaccaccaatgaggcagtt-3′; NR4A3-F, 5′-tgcatgactcaatcagatttgga-3′, NR4A3-R, 5′-agcttggtgtagtcggggt tc-3′; GAPDH-F, 5′-ccagcaagagcacaagaggaa-3′, GAPDH-R, 5′-ggttgagcacagggtactttatt-3′. The relative lncRNA and mRNA expression levels were assessed using the 2^–ΔΔ^ ^Ct^ method.

### Gene Ontology and KEGG Pathway Analysis

To predict the functions of the DE mRNAs correlated with DE lncRNAs, GO ontology^1^ and KEGG (Kyoto Encyclopedia of Genes and Genomes)^2^ pathway analysis were performed as previously described (Zhao et al., 2015; Chen R. et al., 2016).

### LncRNA–mRNA Network Analysis

To explore the interactions between the DE lncRNAs and mRNAs, we performed a network analysis based on a correlation analysis of the DE lncRNAs and mRNAs as previously described (Pujana et al., 2007). The lncRNA–mRNA network was constructed using Cytoscape software (The Cytoscape Consortium, San Diego, CA, United States).

### Cell Culture and Treatment

Human SH-SY5Y cells were cultured in 1:1 mixture of Dulbecco’s Modified Eagle Medium and F12 medium (DMEM/F12; Gibco, Thermo Fisher Scientific, Suzhou, China) supplemented with 10% fetal bovine serum (FBS; Gibco Life Technologies, Grand Island, United States). Lentivirus were packaged in HEK293T cells by transfecting cells with the lentiviral plasmid harboring AC131056.3-001, HOTAIRM1, or the empty vector EX-NEG-Lv201 (GeneCopoeia, Rockville, MD, United States) using Lipofectamine 3000 transfection reagent (Invitrogen, Thermo Fisher Scientific, Carlsbad, CA, United States). Media was changed after 6–8 h of transfection and virus were collected after 48 h. SH-SY5Y cells were infected with the virus and selected with 4 mg/ml puromycin for 48 h to establish stably transfected cell lines. Cells were treated with 6-OHDA (Sigma-Aldrich, St. Louis, MO, United States) at a final concentration of 20 μM for 24 h.

Human monocytic THP-1 cells were cultured in RPMI-1640 medium (Gibco, Thermo Fisher Scientific, Suzhou, China) supplemented with 10% fetal bovine serum (FBS; Gibco Life Technologies, Grand Island, United States) at 37°C and 5% CO_2_. The cells were harvested approximately 3 days later when confluent and seeded in 6-well plates at a concentration of 1.5 × 10^6^ cells per well and were stimulated with a combination of lipopolysaccharide (LPS, 1000 ng/ml) plus interferon-gamma (IFN-γ, 30 ng/ml) (Brown et al., 2014). After 24 h incubation, 1 ml cell-free supernatant as well as 1 ml DMEM/F12 containing 10% FBS were added to SH-SY5Y cells in 6 wells for 72 h. The cells had been plated 24 h earlier at a concentration of 1 × 10^6^ cells per well.

### Cell Viability Assay

Cell viability was detected using the MTS assay system (CellTiter 96 Aqueous One Solution Cell Proliferation Assay, Promega, Madison, WI, United States). Briefly, SH-SY5Y cells stably overexpressing AC131056.3-001, HOTAIRM1, or negative control plasmid were treated with or without 20 μM 6-OHDA for 24 h. Medium was discarded after incubation for 24 h and replaced with 120 μl of medium containing 20 μl of MTS reagent per well and incubated at 37°C for 3 h. The absorbance at 490 nm was measured using an ELX800 microplate reader (Bio-Tek, Winoosk, VT, United States). The values of absorbance were calculated as percentage of the control group.

### Flow Cytometry Assay

Cell apoptosis was analyzed using the Annexin V-APC/7-AAD apoptosis detection kit (Keygen, Jiangsu, China). Briefly, SH-SY5Y cells stably overexpressing AC131056.3-001, HOTAIRM1, or negative control plasmid were treated with or without 20 μM 6-OHDA for 24 h. Then, both attached and detached cells were harvested and resuspended with 200 μl of binding buffer, and Annexin V-APC and 7-AAD were added to the mixtures respectively. After staining for 15 min in the dark, flow cytometry was performed with FACSCalibur (Becton, Dickinson and Company, United States). Cells that were negative for PI and positive for Annexin V were identified as early apoptotic cells, and cells that were positive for PI and Annexin V were identified as late apoptotic cells.

### Statistical Analysis

We analyzed data using Prism version 7.0 (GraphPad Software, La Jolla, CA, United States). For normally distributed data, we calculated means ± SD, and we used parametric tests (independent unpaired Student’s *t*-test) to compare between groups. For non-normally distributed data, we calculated medians (with interquartile ranges) and used non-parametric tests (Mann–Whitney test). We analyzed correlation using Spearman’s rank order correlation analysis. FC and Student’s *t*-test were used to analyze the statistical significance of the microarray results. Values of *p* < 0.05 were considered statistically significant.

## Results

### Distinct lncRNA and mRNA Expression Profiles in Circulating Leukocytes Between PD Patients and Healthy Control Subjects

**FIGURE 1:**
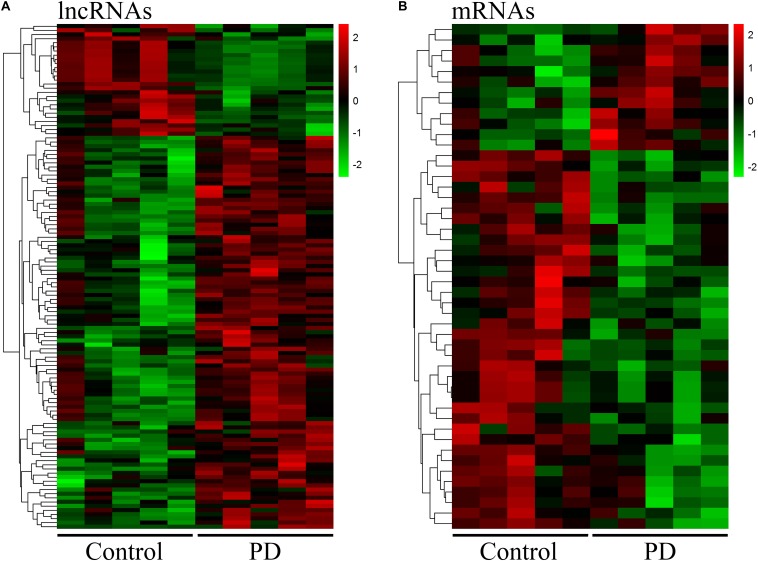
Distinct expression profile of lncRNA and mRNA in circulating leukocytes between PD and healthy controls. The heat map and hierarchical clustering of all differentially expressed lncRNAs **(A)** and mRNAs **(B)** (FC ≥ 2.0) in circulating leukocytes between PD and healthy controls. Each row represents one lncRNA or mRNA and each column represents one subject. The relative expression levels of lncRNA or mRNA are represented by color. Red color denotes high expression levels while green color denotes low expression levels. The numbers in the color scale represent fold changes. *n* = 5 for PD patients, and *n* = 5 for healthy controls. Two-tailed unpaired Student’s *t*-test, *p* < 0.05.

Neuroinflammation induced by peripheral immune cells play a crucial role in the pathogenesis of PD (Wang et al., 2015). We used microarray analysis to profile lncRNA and mRNA expression in circulating leukocytes from PD patients (*n* = 5) and healthy control subjects (*n* = 5). We identified 122 DE lncRNAs in circulating leukocytes of PD patients compared to healthy controls. In the DE lncRNAs, 95 were upregulated while 27 were downregulated (Figure 1A). The top 20 DE lncRNAs were listed in Supplementary Table 1. Meanwhile, we identified 48 DE mRNAs in circulating leukocytes between PD patients and healthy controls. In these DE mRNAs, 12 were upregulated while 36 were downregulated (Figure 1B). The top 20 DE mRNAs were shown in Supplementary Table 2. Our array data were uploaded in Gene Expression Omnibus (GEO) datasets (GSE133347).

**FIGURE 2:**
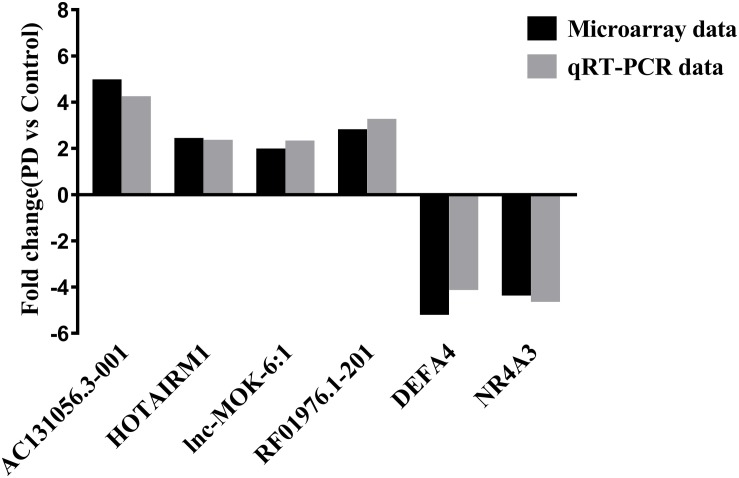
Validation of some of the differentially expressed lncRNAs and mRNAs. The upregulation of the lncRNAs AC131056.3-001, HOTAIRM1, lnc-MOK-6:1, RF01976.1-201, and the downregulation of the mRNA NR4A3 and DEFA4 in circulating leukocytes of PD patients (*n* = 5) compared to healthy controls (*n* = 5) were confirmed using quantitative PCR.

We selected 3 DE lncRNAs (AC131056.3-001, lnc-MOK-6:1, and RF01976.1-201) and 2 DE mRNAs (*DEFA4* and *NR4A3*) from the top 20 DE lncRNAs and mRNAs for validation by using quantitative PCR, respectively. Moreover, we selected HOTAIRM1 for validation because it was implicated in neurogenesis (Lin et al., 2011). Consistent with the microarray data, the expression of the four lncRNAs was upregulated, and the expression of the two mRNAs was downregulated in the PD patients compared with the healthy controls (Figure 2).

### LncRNA–mRNA Network Analysis

**FIGURE 3:**
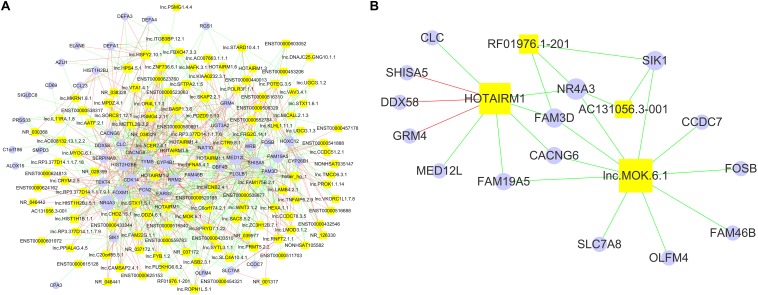
The network of significantly correlated DE lncRNAs and mRNAs. **(A)** The positive correlations among 122 DE lncRNAs (yellow squares) and 48 DE mRNAs (purple closed circles) are represented by red lines, and the negative correlations were represented by green lines. The number of correlations between a specific lncRNA or mRNA and others are represented by the size of the yellow squares and purple closed circles, respectively. **(B)** The network of lncRNAs AC131056.3-001, HOTAIRM1, lnc-MOK-6:1, and RF01976.1-201 and their correlated mRNAs. Non-parametric Spearman correlation analysis was performed. The criteria of *p* < 0.05 and *r* ≥ 0.8 were used to define the positive correlations; and *p* > 0.05 and *r* ≤ −0.8 for negative correlations.

Since lncRNAs play essential roles in gene expression (Quinn and Chang, 2016), the DE lncRNA identified in our array data may affect the expression of the DE mRNA. We analyzed the correlations between the DE lncRNAs and mRNAs from the array data by performing Spearman correlation analysis. Using the criteria of *r* ≥ 0.8 or ≤−0.8 and *p* < 0.05, there were 714 significant correlations among all of the 122 DE lncRNAs and 48 DE mRNAs (Figure 3A). Specifically, we constructed an interaction network of the DE mRNAs correlated with the four lncRNAs (AC131056.3-001, HOTAIRM1, lnc-MOK-6:1, and RF01976.1-201), which were upregulated in PD patients in both microarray and quantitative PCR data (Figure 3B). NR4A subfamily is crucial for the differentiation and maintenance of meso-diencephalic dopaminergic neurons, and protects them from inflammation-induced death (Saucedo-Cardenas et al., 1998; Kadkhodaei et al., 2009; Saijo et al., 2009). Interestingly, we found that NR4A3, a member of NR4A subfamily, was negatively correlated with all of the four DE lncRNAs (Figure 3B). As shown in the Figure 3B, the expression of nine genes was correlated with HOTAIRM1. We examined the mRNA expression of the nine genes in HOTAIRM1-overexpressing SH-SY5Y cells after 6-OHDA treatment. We confirmed that *CLC* and *MED12L* were downregulated whereas *DDX58* was upregulated in the HOTAIRM1-overexpressing SH-SY5Y cells compared with cells transfected with control vector. *CACNG6* and *NR4A3* mRNA tend to be decreased, but the difference was not significant (Supplementary Figure 1). Our data suggest that the DE lncRNAs may contribute to PD pathogenesis by regulating the expression of PD-related genes.

### Gene Enrichment and KEGG Pathway Analyses

**FIGURE 4:**
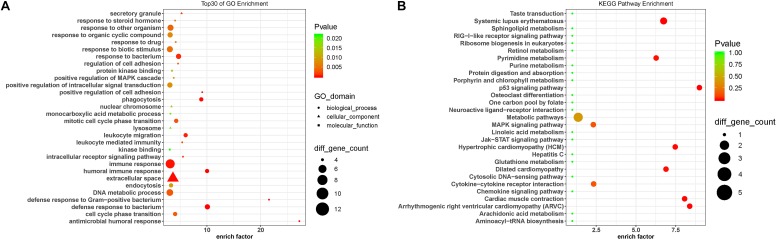
Gene Ontology and KEGG pathway analysis of the DE mRNAs correlated with the DE lncRNAs. **(A)** Top 30 GO terms for the mRNAs correlated with the differentially expressed lncRNAs between the circulating leukocytes of PD patients and healthy controls. **(B)** KEGG pathways analysis of the mRNAs correlated with the differentially expressed lncRNAs between the circulating leukocytes of PD patients and healthy controls. Fisher test, *p* < 0.05.

To predict the functions of the DE lncRNAs, we employed a previously described method (Guttman and Rinn, 2012). Briefly, we conducted a functional enrichment analysis of the mRNAs correlated with the DE lncRNAs. The enriched functional terms were used as the predicted functional terms for lncRNA. Immune dysregulation and inflammation response are involved in PD pathophysiology (Chen L. et al., 2016). The GO terms enriched for the DE mRNAs were related with immune and inflammatory response, including immune response (GO:0006955), leukocyte-mediated immunity (GO:0002443), leukocyte migration (GO:0050900), and positive regulation of MAPK cascades (GO:0043410) (Figure 4A). This suggests that the DE lncRNAs and mRNAs may contribute to PD pathogenesis by affecting immune response. Moreover, the KEGG pathway analysis revealed that the DE mRNAs were enriched in MAPK signaling pathway and Jak-STAT signaling pathway (Figure 4B), which were involved in the pathogenesis of PD by promoting dopaminergic neuron apoptosis (Wang et al., 2012; Zhu et al., 2017).

### Validation of the Four Upregulated lncRNAs in More PD Patients and Healthy Controls

**FIGURE 5:**
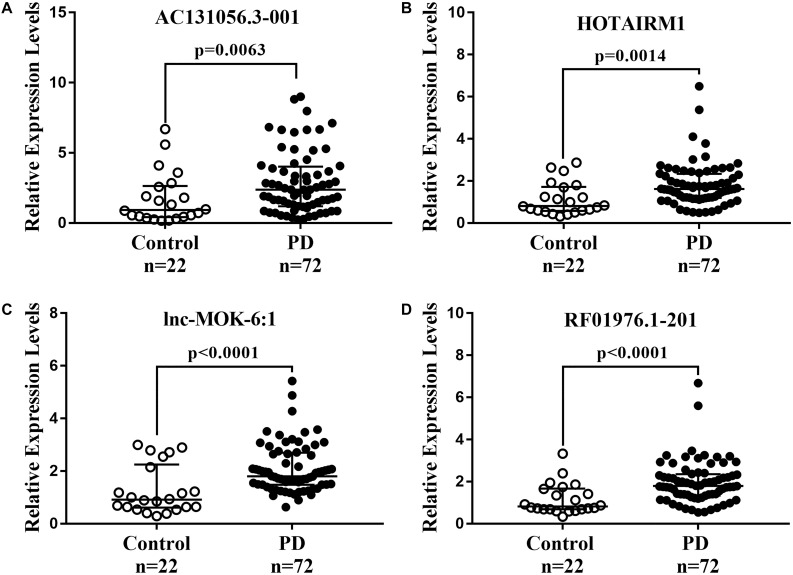
Further confirmation of four upregulated lncRNAs in 72 PD patients and 22 healthy controls. The expression of AC131056.3-001 **(A)**, HOTAIRM1 **(B)**, lnc-MOK-6:1 **(C)**, and RF01976.1-201 **(D)** in circulating leukocytes of PD patients (*n* = 72) and healthy controls (*n* = 22) were determined by quantitative PCR. LncRNAs relative expression levels were expressed as 2^–ΔΔCT^ and relative to the median value for healthy controls (two-tailed Mann–Whitney test). The bar represents median with interquartile range.

**FIGURE 6:**
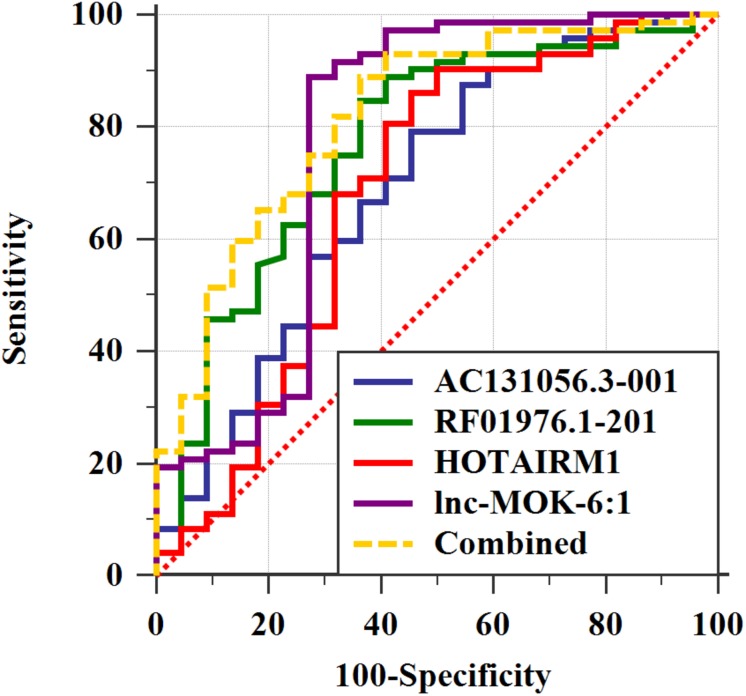
The four upregulated lncRNAs as potential biomarkers for PD diagnosis. ROC curve analyses of lncRNA AC131056.3-001, HOTAIRM1, lnc-MOK-6:1, RF01976.1-201, and the combination of them for diagnosis of PD. *n* = 72 for PD patients, and *n* = 22 for healthy control subjects. Statistical differences between different ROC curves. AUC, area under the ROC curve; ROC, receiver operating characteristic.

We further determined the expression of AC131056.3-001, HOTAIRM1, lnc-MOK-6:1, and RF01976.1-201 by quantitative PCR in 72 PD patients and 22 healthy controls. Consistently, the expression of these four lncRNAs was significantly higher in PD patients compared with controls (Figures 5A–D). To explore whether these lncRNAs could be used as biomarkers for PD, ROC analysis was performed for the four DE lncRNA (Figure 6). The area under the ROC curve (AUC), 95% confidence interval, *p*-value, sensitivity, and specificity of them are shown in Supplementary Table 3. The results indicate that these upregulated lncRNAs may be potential biomarkers for PD diagnosis.

### Upregulation of lncRNAs in Human Monocytic and Neuroblastoma Cell Lines

**FIGURE 7:**
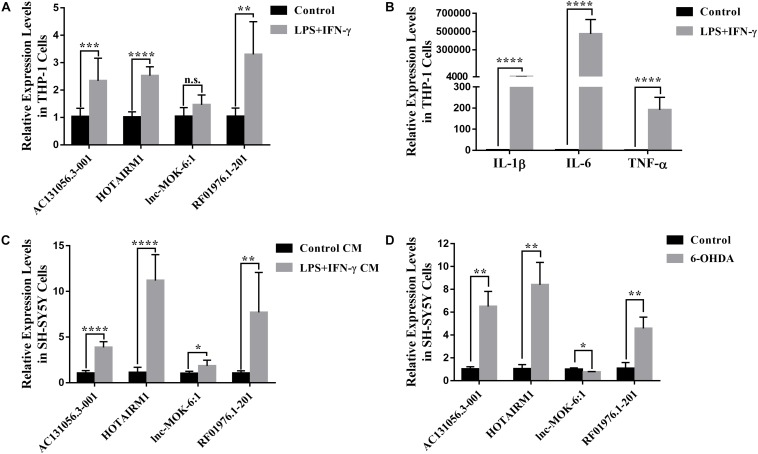
Upregulation of lncRNAs in human monocytic and neuroblastoma cell lines. **(A)** The altered expression levels of AC131056.3-001, HOTAIRM1, and RF01976.1-201 in THP-1 cells after the stimulation of LPS and IFN-γ. **(B)** The increased expression of inflammatory cytokines IL-1β, IL-6, and TNF-α in THP-1 cell stimulated with LPS and IFN-γ. **(C)** The expression of the four lncRNAs in SH-SY5Y cells treated with conditioned culture medium from LPS- and IFN-γ-stimulated THP-1 cells was measured by quantitative PCR. **(D)** The altered expression of the four lncRNAs in SH-SY5Y cells after the treatment of 6-OHDA. Data are mean ± SD, **(A–C)** *n* = 6 wells per group; **(D)** *n* = 3 wells per group. ^∗^*p* < 0.05, ^∗∗^*p* < 0.01, ^∗∗∗^*p* < 0.001, ^****^p < 0.0001. Two-tailed unpaired Student’s *t*-test.

Since the four upregulated lncRNAs were identified in circulating leukocytes of PD patients, we next examined their expression in THP-1 cells, a human monocytic cell line. We found that the expression of AC131056.3-001, HOTAIRM1, and RF01976.1-201 were significantly increased in THP-1 cells stimulated with LPS and IFN-γ compared to control cells (Figure 7A). Consistent with previous reports (Singh et al., 2016), the expression of inflammatory cytokines IL-1β, IL-6, and TNF-α was increased in THP-1 cells stimulated with LPS and IFN-γ (Figure 7B). Interestingly, conditioned culture medium from LPS and IFN-γ stimulated THP-1 cell culture but not from control cells significantly increased the expression of the four lncRNAs in SH-SY5Y cells, a neuroblastoma cell line expressing dopaminergic markers (Figure 7C). However, direct stimulation with LPS and IFN-γ did not alter the expression of the four lncRNAs (Supplementary Figure 2). Our data suggests that circulating leukocytes may alter the expression of lncRNAs in neurons in a paracrine manner. Further, HOTAIRM1 overexpression led to a significant increase of IL-1β and TNF-α expression in LPS- and IFN-γ-stimulated THP-1 cells. This indicates that HOTAIRM1 upregulation may be responsible for the inflammatory cytokine expression in leukocytes (Supplementary Figure 3). We also examined the expression of the four lncRNAs in SH-SY5Y cells stimulated with 6-OHDA, an *in vitro* system often used in PD study (Sever et al., 2016). Consistent with our findings in PD patients, AC131056.3-001, HOTAIRM1, and RF01976.1-201 expression were significantly increased in SH-SY5Y cells after 6-OHDA stimulation (Figure 7D). This suggests that the DE lncRNAs are involved in the pathogenesis of PD.

### Overexpression of AC131056.3-001 or HOTAIRM1 Decreases Cell Viability and Increases Apoptosis in SH-SY5Y Cells

**FIGURE 8:**
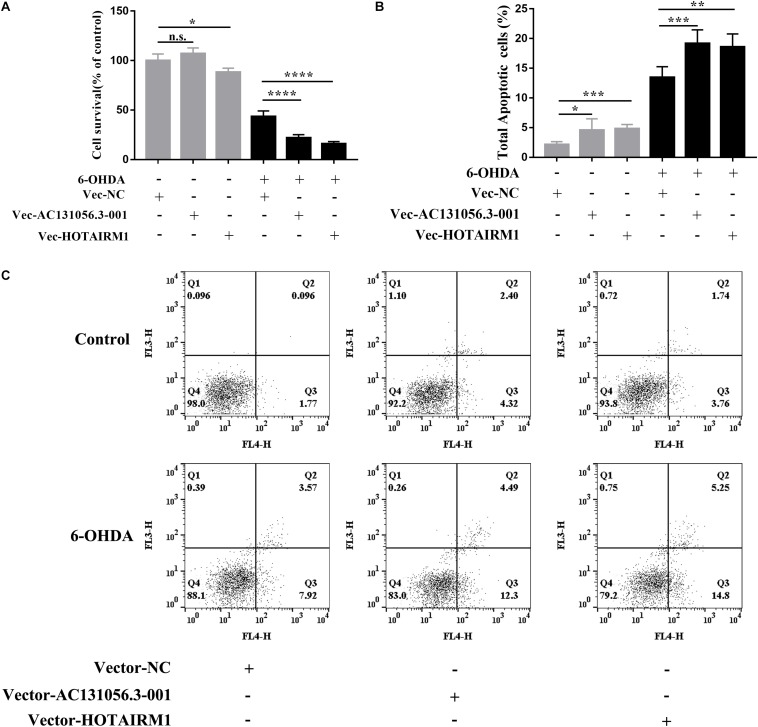
Overexpression of AC131056.3-001 or HOTAIRM1 decreases cell viability and increases apoptosis in SH-SY5Y cells. **(A)** Overexpression of AC131056.3-001 or HOTAIRM1 decreases the viability of SH-SY5Y cells treated with or without 6-OHDA. Cell viability was evaluated using the MTS assay. *n* = 5 wells per group. **(B,C)** Flow cytometry assay showed that overexpression of AC131056.3-001 or HOTAIRM1 promoted the apoptosis of the SH-SY5Y cells treated with or without 6-OHDA. *n* = 6. Data are mean ± SD, ^∗^*p* < 0.05, ^∗∗^*p* < 0.01, ^∗∗∗^*p* < 0.001, ^****^p < 0.0001. Two-tailed unpaired Student’s *t*-test.

PD is characterized by a selective loss of dopaminergic neurons in the substantia nigra pars compacta (Braak et al., 2003). Since both gene enrichment and KEGG pathway analysis suggests that DE lncRNA may be involved in PD through affecting apoptosis of dopaminergic neuron, we next examined whether DE lncRNAs are involved in the cell viability and apoptosis of dopaminergic neuron. We first investigated the role of AC131056.3-001 or HOTAIRM1 in the cell viability of SH-SY5Y cells. SH-SY5Y cells were stably transfected with AC131056.3-001 or HOTAIRM1, respectively. 6-OHDA stimulation induced the expression of AC131056.3-001 or HOTAIRM1, and further enhanced the expression of AC131056.3-001 or HOTAIRM1 in cells transfected with the overexpression vectors of these lncRNAs (Supplementary Figure 4). The viability of SH-SY5Y cells were significantly decreased after treatment with 6-OHDA. Overexpression of AC131056.3-001 or HOTAIRM1 further decreased the viability of SH-SY5Y cells treated with or without 6-OHDA (Figure 8A), whereas knockdown of AC131056.3-001 or HOTAIRM1 partly rescued the reduced viability of 6-OHDA-treated cells (Supplementary Figure 5). Furthermore, we evaluated the effect of AC131056.3-001 or HOTAIRM1 overexpression on the apoptosis of SH-SY5Y cells by flow cytometry. Similar to a previous report (Guo et al., 2005), the apoptosis of SH-SY5Y cells was significantly increased after 6-OHDA treatment.

Overexpression of AC131056.3-001 or HOTAIRM1 increased baseline and the 6-OHDA-induced apoptosis of SH-SY5Y cells, including early apoptosis and late apoptosis (Figures 8B,C). These data suggest that AC131056.3-001 or HOTAIRM1 reduces the cell viability and promotes the apoptosis of dopaminergic neurons.

## Discussion

In the present study, we demonstrated that there were distinct lncRNA and mRNA expression profiles in the circulating leukocytes between PD patients and healthy controls. Strong correlations existed among the 122 DE lncRNAs and the 48 DE mRNAs identified in the array data. The upregulation of four lncRNAs were further confirmed in 72 PD patients compared with 22 healthy controls. The upregulation of the lncRNAs were also observed in human monocytic and neuroblastoma cell lines. For the first time, we demonstrate that AC131056.3-001 or HOTAIRM1 reduced the cell viability and promoted the apoptosis of SH-SY5Y cells.

It has been shown that neuroinflammation and dopamine neurons death induced by circulating immune cells play a crucial role in the pathogenesis of PD (Ji et al., 2007; Brochard et al., 2009). Thus, we profiled the expression of lncRNA and mRNA in circulating leukocytes of PD patients and healthy controls. Using microarray technology, we identified 122 DE lncRNAs and 48 DE mRNAs between PD patients and healthy controls. We summarized the information including the platforms used in the available microarray or RNA-seq data related to PD patients in GEO datasets (Supplementary Table 4). Recently, Chi reported 7 DE lncRNAs and 394 DE mRNAs in PD blood samples by reanalyzing the microarray datasets GSE6613 (Chi et al., 2019). A possible reason for the more identified DE lncRNAs in our study is that the array platform GPL21047 (Agilent-074348 Human lncRNA v6 4 × 180K probe) used in our study focused on the lncRNA expression, whereas the platform GPL96 (Affymetrix Human Genome U133A Array) for GSE6613 focused on mRNA expression. Soreq et al. (2014) also reported that the lncRNA expression profile in leukocytes was widely altered in PD patients before and after deep brain stimulation compared with controls. Moreover, altered lncRNA expression profiles in brain specimen of PD patients was also reported (Kraus et al., 2017).

It was reported that lncRNAs play essential roles in the pathogenesis of PD via regulating gene expression (Liu et al., 2016; Liu and Lu, 2018; Xu et al., 2018). Several studies reported altered mRNA expression profiles of PD blood samples (Scherzer et al., 2007; Jiang et al., 2019). One advantage of our study is that we simultaneously profiled both of the lncRNA and mRNA expression in circulating leukocytes from the same PD patients and healthy controls. We found that as many as 714 significant correlations existed among all of the 122 DE lncRNAs and the 48 DE mRNAs. Notably, our finding that NR4A3 mRNA expression was decreased in PD patients is consistent with another study (Montarolo et al., 2016). Moreover, lncRNA–mRNA network analysis identified that NR4A3 was negatively correlated with four upregulated lncRNAs, AC131056.3, HOTAIRM1, lnc-MOK-6:1, and RF01976.1-201. Further study is required to clarify whether these lncRNAs can regulate the expression of NR4A3.

Neuroinflammation is a key pathophysiological process of PD. Both innate immune response (Chandra et al., 2016) and adaptive immune response (Brochard et al., 2009) are involved in the pathogenesis of PD. LncRNA SNHG1 was reported to promote neuroinflammation in PD via acting as a competing endogenous RNA to upregulate NLRP3 expression (Cao et al., 2018). Interestingly, GO analysis of the DE mRNA identified several GO terms related to immunity and inflammation, including immune response, inflammatory response, leukocyte migration, and cytokine production. Our findings suggest that lncRNA may contribute to PD pathogenesis by regulating the expression of the genes related to immune response and inflammation. Moreover, KEGG pathway analysis showed that the altered lncRNA AC007991.2-201, AC084816.1-207, and HOTAIRM1 were enriched in MAPK signaling pathway, which was implicated in the pathogenesis of PD, including inflammation (Zhang et al., 2016) and cell death (Wang et al., 2012). Moreover, we identified that several DE lncRNAs including HOTAIRM1 were enriched in Jak-STAT signaling pathway, which plays a critical role in PD by promoting neuroinflammation and dopaminergic neuron apoptosis (Qin et al., 2016; Zhu et al., 2017).

Non-invasive biomarkers are attractive in the detection and diagnosis of PD. In the present work, we found that AC131056.3, HOTAIRM1, lnc-MOK-6:1, and RF01976.1-201 were upregulated in circulating leukocytes of PD patients using microarray technology. This was further confirmed in 72 PD patients and 22 healthy control subjects using quantitative PCR. Moreover, ROC analysis revealed that these four lncRNAs were potential biomarkers in the diagnosis of PD.

In our further investigation of the lncRNAs in *in vitro* systems of PD, we demonstrated that the lncRNAs were upregulated in THP-1 cells after inflammatory stimuli and in SH-SY5Y cells treated with THP-1 cell conditioned culture medium or 6-OHDA. These observations suggest that circulating leukocytes can regulate the lncRNA expression in neurons, and the lncRNAs identified in circulating leukocytes may also be upregulated in neurons and involved in the pathogenesis of PD. It was reported that HOTAIRM1 enhanced the autophagy in acute promyelocytic leukemia (Chen et al., 2017) and contributed to the suppression of colorectal cancer (Wan et al., 2016). However, the role of HOTAIRM1 in PD remains unknown. For the first time, we reported that overexpression of AC131056.3-001 or HOTAIRM1 promoted the apoptosis of SH-SY5Y cells. This suggests that AC131056.3-001 or HOTAIRM1 may contribute to PD by promoting the apoptosis of dopaminergic neuron. However, the mechanism by which AC131056.3-001 or HOTAIRM1 promotes the apoptosis of dopaminergic neurons requires further study. Another limitation of our study is the lack of direct evidence for the upregulation of these lncRNAs in the neurons of PD patients.

Taken together, there were distinct expression profiles of lncRNA and mRNA in circulating leukocytes between PD patients and healthy controls. The dysregulated lncRNAs such as HOTAIRM1 and AC131056.3-001 may contribute to the pathogenesis of PD by promoting the apoptosis of dopaminergic neurons. These dysregulated lncRNA may be potential targets for PD therapy.

## Data Availability Statement

The datasets generated for this study can be found in the Gene Expression Omnibus; https://www.ncbi.nlm.nih.gov/geo/query/acc.cgi?acc=GSE133347.

## Ethics Statement

The studies involving human participants were reviewed and approved by the Ethics Committee of Tongji Hospital, Huazhong University of Science and Technology. The patients/participants provided their written informed consent to participate in this study.

## Author Contributions

ZX designed the research, conceived the manuscript, had primary responsibility for writing, and edited and revised the manuscript. YF, QY, CG, HG, ZM, JL, and XY performed the experiments. YF, QY, SZ, and ZX analyzed the data. YF and ZX prepared the figures and drafted the manuscript.

## Conflict of Interest

The authors declare that the research was conducted in the absence of any commercial or financial relationships that could be construed as a potential conflict of interest.

## Footnotes

1 http://geneontology.org/

2 http://www.genome.jp/kegg/

## Abbreviations

6-OHDA: 6-Hydroxydopamine hydrochloride
AUC: area under the curve
CNS: central neuron system
DE: differentially expressed
FC: fold change
GO: Gene Ontology
KEGG: Kyoto Encyclopedia of Genes and Genomes
lncRNA: long non-coding RNA
MAPK: mitogen-activated protein kinase
PD: Parkinson’s disease
ROC: receiver operating characteristic.
