# Supplementary material for: Dysregulated Long Non-coding RNAs in Parkinson’s Disease Contribute to the Apoptosis of Human Neuroblastoma Cells
Source: Front Neurosci. 2019 Dec 13;13:1320. doi: 10.3389/fnins.2019.01320 (PMC6923663; doi:10.3389/fnins.2019.01320)
Supplement: Supplementary file 1 [file Data_Sheet_1.pdf]

SUPPLEMENTARY MATERIAL

SUPPORTING INFORMATION

METHODS

Knockdown of lncRNAs via transfecting with siRNA in SH-SY5Y cells.

The expression of AC131056.3-001 or HOTAIRM1 was knockdown via transfection of small interfering RNAs (siRNAs) (RiboBio, Guangzhou, China) in SH-SY5Y cells. The sequences of siRNA against human AC131056.3-001 or HOTAIRM1 were 5'-CCGTTCAATGAAAGATGAA-3' and 5'-CCGTTCAATGAAAGATGAA-3', respectively. Cells were transfected using Lipofectamine 3000 transfection reagent (Invitrogen, Thermo Fisher Scientific, Carlsbad, US) with 100 nM of oligonucleotides according to the manufacturer's instruction.

SUPPLEMENTARY MATERIAL TABLES

Supplementary Material Table 1. Top 20 aberrantly expressed lncRNAs in microarray analysis

SeqID	Fold Change	P value	Chr	Strand
ENST00000506329	6.9508	0.0057	chr11	+
ENST00000615128	4.8666	0.0092	chr17	+
lnc-CTR9-8:1	4.2787	0.0358	chr11	+
AC131056.3-001	4.1377	0.0263	chr17	+
ENST00000541888	4.0971	0.0214	chr12	+
lnc-HIST1H2BJ-5:1	3.9947	0.0034	chr6	-
lnc-AC008132.13.1-2:2	3.8236	0.0414	chr22	+
lnc-MAFK-3:1	3.7948	0.0391	chr7	+
lnc-ACER2-4:1	3.3282	0.0411	chr9	+
RF01976.1-201	3.1542	0.0296	chr7	+
lnc-MKRN1-8:1	2.9473	0.0041	chr7	-
lnc-SORCS1-12:1	2.8420	0.0185	chr10	-
lnc-ITGB3BP-12:1	2.7338	0.0461	chr1	-
lnc-LAMB4-2:1	2.7098	0.0329	chr7	-
lnc-MYOC-6:1	2.6890	0.0285	chr1	-
NR_038328	0.3699	0.0061	chr21	-
lnc-RNFT2-1:1	0.3519	0.0164	chr12	+
ENST00000520185	0.3431	0.0002	chr8	+
lnc-LMOD3-1:2	0.3322	0.0263	chr3	-
lnc-MICALL2-1:3	0.2550	0.0349	chr7	-

SeqID: lncRNA name. P value: p value calculated from unpaired t-test. Fold Change: the absolute ratio (no log scale) of normalized intensities between two groups (PD vs healthy control). Chr: chromosome number which lncRNA is transcribed. Strand: the strand of chromosome which lncRNA is transcribed; '+' is sense strand of chromosome, '−' is antisense strand of chromosome.

Supplementary Material Table 2. Top 20 aberrantly expressed mRNAs in microarray analysis

SeqID	Gene Symbol	Fold Change	P value	Chr	Strand
NM_145663	DBF4B	2.7279	0.0368	chr17	+
NM_001286559	TEKT4	0.3978	0.0238	chr2	+
NM_173354	SIK1	0.3954	0.0019	chr21	-
NM_019885	CYP26B1	0.3867	0.0144	chr2	-
NM_014442	SIGLEC8	0.3696	0.0413	chr19	-
NM_005064	CCL23	0.3688	0.0293	chr17	-
NM_018667	SMPD3	0.3607	0.0387	chr16	-
NM_001828	CLC	0.3572	0.0052	chr19	-
NM_152891	PRSS33	0.3456	0.0439	chr16	-
NM_002922	RGS1	0.3377	0.0126	chr1	+
NM_004084	DEFA1	0.3341	0.0457	chr8	-
NM_012244	SLC7A8	0.3241	0.0009	chr14	-
NM_005217	DEFA3	0.2992	0.0398	chr8	-
NM_006418	OLFM4	0.2974	0.0058	chr13	+
NM_004084	DEFA1	0.2923	0.0363	chr8	-
NM_004627	WRB	0.2895	0.0157	chr21	+
NM_006732	FOSB	0.2638	0.0371	chr19	+
NM_001925	DEFA4	0.2496	0.0355	chr8	-
NM_173200	NR4A3	0.2213	0.0072	chr9	+
NM_052943	FAM46B	0.1872	0.0022	chr1	-

SeqID: mRNA name. Gene Symbol: gene name. P value: p value calculated from unpaired t-test. Fold Change: the absolute ratio (no log scale) of normalized intensities between two groups (PD vs healthy control). Chr: chromosome number which mRNA is transcribed. Strand: the strand of chromosome which mRNA is transcribed; '+' is sense strand of chromosome, '–' is antisense strand of chromosome.

Supplementary Material Table 3. The area under the ROC curve (AUC), 95% Confidence interval (CI), p value, sensitivity and specificity of the four lncRNAs and the combine of all of them in PD patients (n=72)

lncRNA	AUC	95% CI	P value	Sensitivity	Specificity
AC131056.3-001	0.691	0.588-0.783	0.0063	79.17	54.55
RF01976.1-201	0.77	0.671-0.850	<0.0001	84.72	63.64
HOTAIRM1	0.683	0.579-0.775	0.0168	86.11	54.55
lnc-MOK-6:1	0.776	0.678-0.855	0.0002	88.89	72.73
Combined	0.817	0.724-0.889	<0.0001	88.89	63.64

ROC, receiver operating characteristic; Combine, the combination of lncRNA AC131056.3-001, HOTAIRM1, lnc-MOK-6:1 and RF01976.1-201.

Supplementary Material Table 4. The information for the available microarray or RNA-seq data related to PD patients in GEO

GSE Number	Time of Open to Publics	Microarray/ RNA-Seq	Samples	HC	PD	Platforms
GSE6613	2006/12/30	microarray	Whole blood	22	50	GPL96 [HG-U133A] Affymetrix Human Genome U133A Array
GSE99039	2017/3/20	microarray	Whole blood	233	205	GPL570 [HG-U133_Plus_2] Affymetrix Human Genome U133 Plus 2.0 Array
GSE54536	2014/1/31	microarray	Whole blood	5	5	GPL10558 Illumina HumanHT-12 V4.0 expression beadchip
GSE72267	2015/10/29	microarray	Whole blood	20	40	GPL571 [HG-U133A_2] Affymetrix Human Genome U133A 2.0 Array
GSE57475	2015/6/1	microarray	Whole blood	49	93	GPL6947 Illumina HumanHT-12 V3.0 expression beadchip
GSE8397	2008/1/1	microarray	Substantia Nigra	24	15	GPL96 [HG-U133A] Affymetrix Human Genome U133A Array GPL96 [HG-U133A] Affymetrix Human Genome U133B Array
GSE7621	2007/6/14	microarray	Substantia Nigra	9	16	GPL570 [HG-U133_Plus_2] Affymetrix Human Genome U133 Plus 2.0 Array
GSE68719	2016/1/15	mRNA-Seq	BA9 brain tissue	44	29	GPL11154 Illumina HiSeq 2000 (Homo sapiens)
GSE20292	2010/3/12	microarray	Substantia Nigra	18	11	GPL96 [HG-U133A] Affymetrix Human Genome U133A Array
GSE42608	2014/7/16	RNA-Seq	Blood Leukocytes	3	3	GPL9442 AB SOLiD System 3.0 (Homo sapiens)
GSE19587	2010/8/19	microarray	ION and DMNV	5	6	GPL571 [HG-U133A_2] Affymetrix Human Genome U133A 2.0 Array

Abbreviations: GEO, Gene Expression Omnibus; PD, Parkinson's disease; HC, healthy control; DE, differentially expressed; ION, Inferior Olivary Nucleus; DMNV, Dorsal Motor Nucleus of the Vagus; DBS, deep brain stimulation; Stim, stimulation.

SUPPLEMENTARY FIGURES

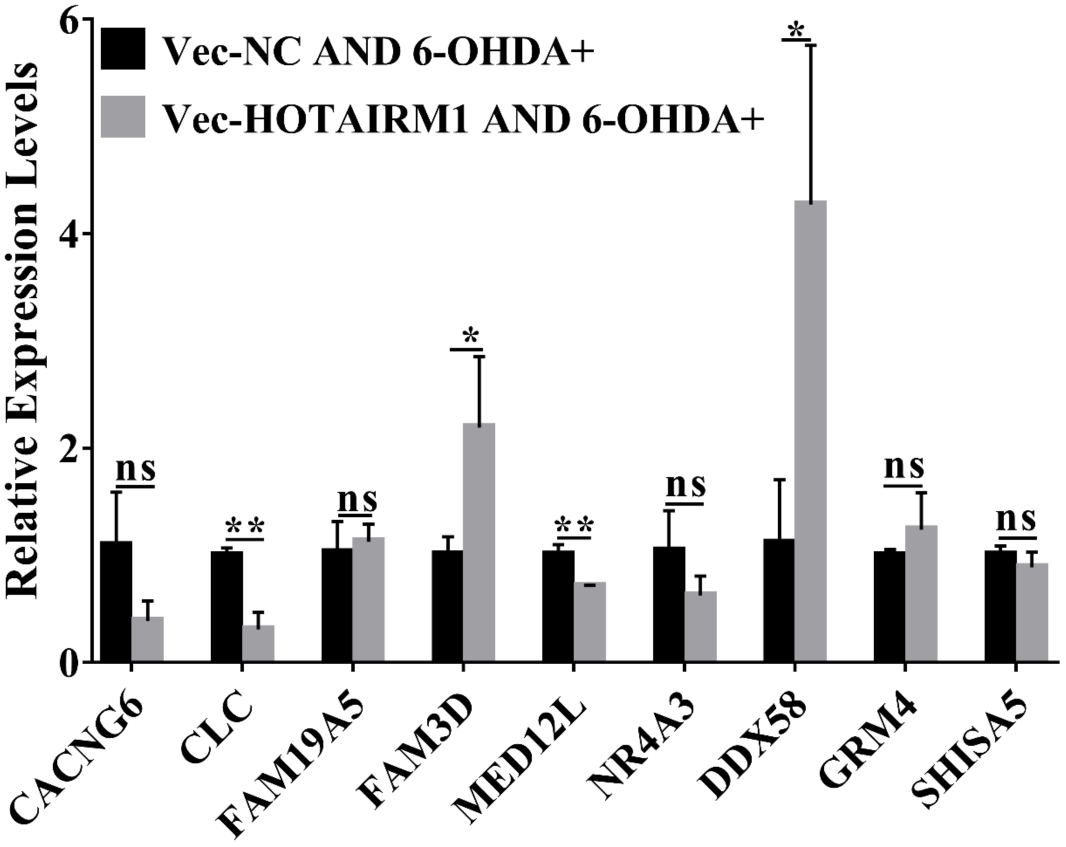

Supplementary Material Figure 1. Validation of the genes correlated with HOTAIRM1 expression in SH-SY5Y cells. The mRNA expression of the nine genes (*CACNG6, CLC, FAM19A5, FAM3D, MED12L, NR4A3, DDX58, GRM4, SHISA5*) that correlated with HOTAIMR1 expression were examined by using qPCR in HOTAIRM1-overexpressing SH-SY5Y cells after 6-OHDA treatment. n = 3. Data are mean ± SD, *p < 0.05, ** p<0.01, n.s., no significance. Two-tailed unpaired Student's t test.

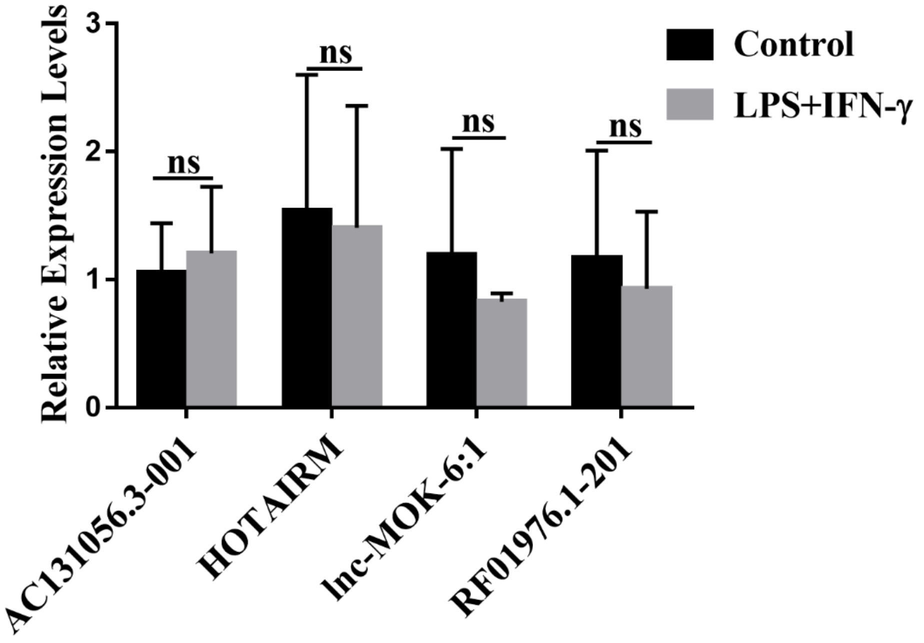

Supplementary Material Figure 2. LPS and IFN-γ stimulation did not alter the expression of the four lncRNAs. The expression of the lncRNAs AC131056.3-001, HOTAIRM1, lnc-MOK-6:1 and RF01976.1-201 in SH-SY5Y cells were determined by using qPCR after the direct stimulations of LPS and IFN-γ. n = 3-6. Data are mean ± SD, n.s., no significance. Two-tailed unpaired Student's t test.

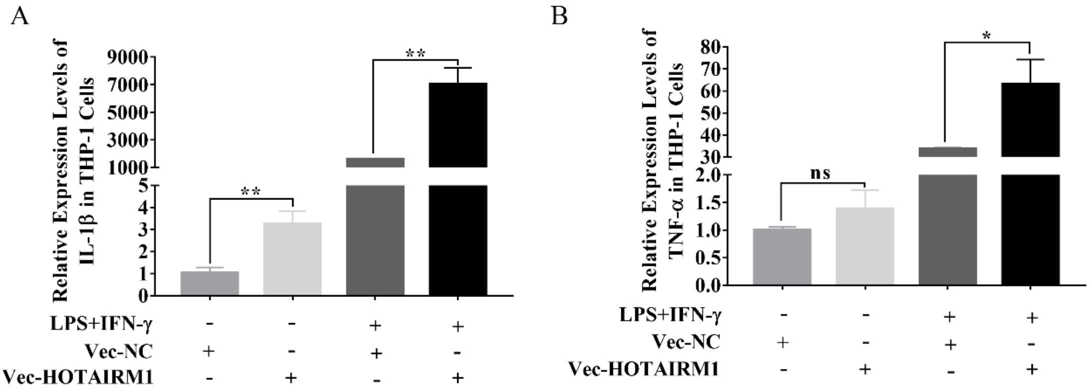

Supplementary Material Figure 3. HOTAIRM1 overexpression induced IL-1β and TNF-α expression in THP-1 cells. The expression of IL-1β and TNF-α were determined by using qPCR in control and HOTAIRM1-overexpressing THP-1 cells with or without the treatment of LPS and IFN-γ. n = 3. Data are mean ± SD, *$p < 0.05$, ** $p<0.01$, n.s., no significance. Two-tailed unpaired Student's t test.

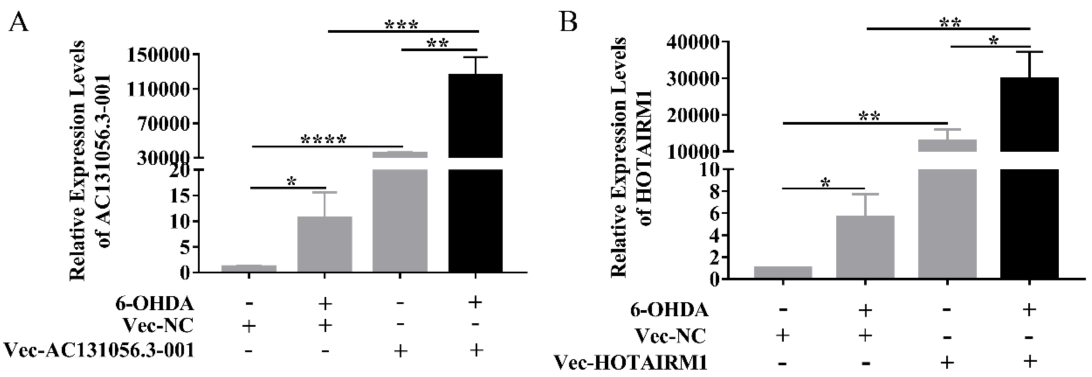

Supplementary Material Figure 4. 6-OHDA stimulation induced the expression of AC131056.3-001 or HOTAIRM1 in SH-SY5Y cell. The expression of AC131056.3-001 or HOTAIRM1 were determined by using qPCR in SH-SY5Y cells after 6-OHDA stimulation with or without overexpression of the two lncRNAs. n = 3. Data are mean ± SD, *p < 0.05, ** p<0.01, *** p < 0.001, **** p<0.0001. Two-tailed unpaired Student's t test.

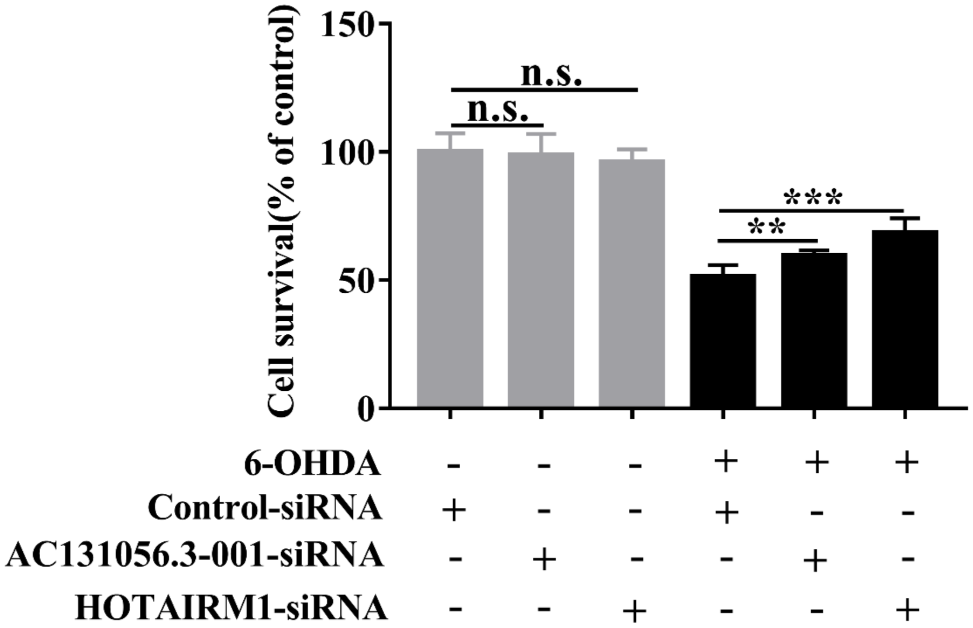

Supplementary Material Figure 5. Knockdown of AC131056.3-001 or HOTAIRM1 partly rescued the reduced viability of 6-OHDA-treated SH-SY5Y cells. Cell viability was evaluated using the MTS assay. n = 5 wells per group. Data are mean ± SD, n.s., no significance**,** ** $p<0.01$, *** $p < 0.001$. Two-tailed unpaired Student's t test.
